# Functional interaction between mouse erbB3 and wild-type rat c-*neu *in transgenic mouse mammary tumor cells

**DOI:** 10.1186/bcr1281

**Published:** 2005-07-06

**Authors:** Aeree Kim, Bolin Liu, Dalia Ordonez-Ercan, Kathy M Alvarez, Lynn D Jones, Christine McKimmey, Susan M Edgerton, XiaoHe Yang, Ann D Thor

**Affiliations:** 1 Department of Pathology and College of Medicine, Oklahoma University Health Sciences Center (OUHSC), Oklahoma City, OK, USA; 2 Department of Pathology, College of Medicine, Korea University, Seoul, Korea

## Abstract

**Introduction:**

Co-expression of several receptor tyrosine kinases (RTKs), including erbB2 and erbB3, is frequently identified in breast cancers. A member of the RTK family, the kinase-deficient erbB3 can activate downstream signaling via heterodimer formation with erbB2. We studied the expression of RTK receptors in mammary tumors from the wild-type (wt) rat c-*neu *transgenic model. We hypothesized that physical and functional interactions between the wt rat *neu/ErbB2 *transgene and mouse *ErbB3*-encoded proteins could occur, activating downstream signaling and promoting mammary oncogenesis.

**Methods:**

Immunohistochemical and Western blot analyses were performed to study the expression of rat c-*neu/*ErbB2 and mouse erbB3 in mammary tumors and tumor-derived cell lines from the wt rat c-*neu *transgenic mice. Co-immunoprecipitation methods were employed to quantitate heterodimerization between the transgene-encoded protein erbB2 and the endogenous mouse erbB3. Tumor cell growth in response to growth factors, such as Heregulin (HRG), epidermal growth factor (EGF), or insulin-like growth factor-1 (IGF-1), was also studied. Post-HRG stimulation, activation of the RTK downstream signaling was determined by Western blot analyses using antibodies against phosphorylated Akt and mitogen-activated protein kinase (MAPK), respectively. Specific inhibitors were then used with cell proliferation assays to study the phosphoinositide-3 kinase (PI-3K)/Akt and MAPK kinase (MEK)/MAPK pathways as possible mechanisms of HRG-induced tumor cell proliferation.

**Results:**

Mammary tumors and tumor-derived cell lines frequently exhibited elevated co-expression of erbB2 and erbB3. The transgene-encoded protein erbB2 formed a stable heterodimer complex with endogenous mouse erbB3. HRG stimulation promoted physical and functional erbB2/erbB3 interactions and tumor cell growth, whereas no response to EGF or IGF-1 was observed. HRG treatment activated both the Akt and MAPK pathways in a dose- and time-dependent manner. Both the PI-3K inhibitor LY 294002 and MEK inhibitor PD 98059 significantly decreased the stimulatory effect of HRG on tumor cell proliferation.

**Conclusion:**

The co-expression of wt rat *neu/ErbB2 *transgene and mouse *ErbB3*, with physical and functional interactions between these two species of RTK receptors, was demonstrated. These data strongly suggest a role for erbB3 in c-*neu *(ErbB2)-associated mammary tumorigenesis, as has been reported in human breast cancers.

## Introduction

The erbB or epidermal growth factor receptor (EGFR) family forms subclass I of the receptor tyrosine kinase (RTK) superfamily. Type I RTKs are expressed by epithelial, mesenchymal and neural tissues to regulate cell proliferation, differentiation and other important biological functions critical to species development [1]. Dysregulated expression of erbB receptors or mutational events thereof have been implicated in diverse types of human cancers [1,2]. Members of the family include: ErbB1 (also known as EGFR), ErbB2 (also known as Her-2 or *neu*), ErbB3 (or Her-3) and ErbB4 (or Her-4) [3-7]. erbB2 is an orphan receptor whereas other family members directly bind ligands (like the epidermal growth factor (EGF) and transforming growth factor-α (TGF-α) for EGFR, and HRG for erbB3 and erbB4) to initiate intracellular signaling [8].

ErbB2 may be activated via either ligand-dependent heterodimeric, or ligand-independent homodimeric processes. In the former, erbB2 is the preferred heterodimerization partner for other erbB family receptors with bound ligand [9]. In ligand-independent signaling, erbB2 may be upregulated as a result of gene amplification, promoting homodimerization, or be activated through mutational events. *ErbB2 *amplification with enhanced protein expression is noted in approximately one-third of invasive human breast cancers [10]. Selected heterodimers may enhance receptor activation and downstream signaling as compared with homodimers [1,11,12]. Although erbB3 lacks a functional kinase to initiate cell signaling [13,14], the erbB2/erbB3 heterodimer complex is believed to be the most biologically active and pro-tumorigenic form of these receptor complexes [15,16].

The erbB receptors and their respective ligands influence a wide range of cellular processes such as proliferation, maturation, survival, apoptosis and angiogenesis [11,17-19]. In general, activated RTKs add phosphorylated tyrosine residues to downstream signaling molecules, such as the p85 subunit of phosphatidylinositol 3-kinase (PI-3K), Shc and/or Grb2 of the mitogen-activated protein kinase (MAPK) pathway. However, because of the complexity of RTK ligand-dependent and -independent mechanisms, the downstream signaling effects may be highly diverse and interactive. RTK-induced signaling is also influenced by, and may modulate, other molecular factors and signaling pathways.

The *ErbB2 *gene-encoded protein is over-expressed in 25 to 30% of invasive breast and ovarian cancers and has been associated with a poor clinical outcome [20-25].

Evidence of a causal relationship in human breast cancer has been derived from numerous prognostic studies and clinical trials. *In vivo *and *in vitro *model systems including transgenic mouse models support a relationship between erbB2 alterations and mammary tumorigenesis. Overexpression of erbB3 is also frequently reported in erbB2 altered breast, ovarian and bladder cancers [23,26,27]. Human breast cancer cell lines commonly co-overexpress both erbB2 and erbB3, further supporting their role in breast carcinogenesis [2,11].

To investigate the role of RTKs in mammary tumorigenesis, transgenic mice bearing the wild-type (wt) or mutated, activated rat c-*neu *(*ErbB2*) were generated, and have been widely studied [28-31]. Transgenic mice expressing the activated rat c-*neu *(with deletion mutations) bear mammary tumors with elevated co-expression of the mutant c-*neu*/*ErbB2 *and the endogenous mouse *ErbB3*-encoded protein [32]. Functional and physical interactions between these two cross-species receptors have not been reported, although interactions have been widely speculated. Transgenic mice bearing the wt-rat c-*neu*, under control of the mouse mammary tumor virus promoter (MMTV-LTR), typically develop unifocal, well-circumscribed, low-grade tumors after a long latency [29]. In addition to transgene expression and, in some cases, mutation, upregulation of EGFR and p53 have been reported in derived tumors [33,34].

We have used the wt-erbB2 transgenic mouse model to study the effects of exogenous pharmacological or dietary estrogens and anti-estrogens. In particular, we have studied interactions between RTK-associated mammary tumorigenesis and steroid hormones. From the derived mouse tumors, we have established over 150 novel murine cell lines which have proven useful for *in vitro *studies [35,36]. Most tumor-derived cell lines express significant mouse *ErbB3*-encoded protein, in addition to high levels of the rat c-*neu*/*ErbB2 *transgene. These are also typically negative for ERα but show ERβ protein expression. A similar pattern of receptor expression has also been detected in the mouse mammary tumors.

The co-expression of erbB3 with erbB2 in both the activated and wt-*neu*/*ErbB2 *transgenic model systems suggested a biological role for erbB3 in mammary tumor pathogenesis. We hypothesized that physical and functional interactions between these RTK receptors should occur, despite their cross-species molecular structures. Signaling initiated by activated erbB2/erbB3 heterodimers should provide a more potent oncogenic signal than erbB2 homodimers alone. This would require ligand binding, most likely HRG, to activate erbB3. To test this hypothesis, we studied the responsiveness of tumor-derived cell lines to growth factors, including HRG, EGF and insulin-like growth factor-1 (IGF-1); we evaluated the effects of ligand stimulation and heterodimer formation on downstream signaling activation; and we sought evidence of physical interactions between the wt-rat c-*neu*/erbB2 and the endogenous mouse erbB3.

## Materials and methods

### Cells and cell culture

Human breast cancer cell lines SKBR-3 and BT-474 were obtained from the American Type Culture Collection (Rockville, MD, USA) and maintained in DMEM and Ham's F-12 medium (1:1, v/v) (Invitrogen Corp, Grand Island, NY, USA) supplemented with 10% FBS (Invitrogen Corp). These cell lines were cultured in a 37°C humidified atmosphere containing 95% air and 5% CO_2 _and were split twice a week. These human breast cancer cells were used primarily as controls.

### Establishment of novel, mouse mammary tumor cell lines

Mammary tumors were obtained from the transgenic mice by surgical removal immediately following euthanasia, according to our approved IACUC protocol. The histological pattern and tumor diagnoses were confirmed by microscopic analysis. These methods have been previously described in detail [35], although the specific cell lines described in this work have not been previously published. In brief, solid tumor tissue was transferred into a tissue culture dish containing PBS. After removal of mammary fat and connective tissues, tumors were minced into small pieces and treated with 0.25% trypsin-EDTA (Invitrogen Corp) at 37°C for 30 min. Cells were subsequently centrifuged at 1,200 rpm for 5 min. After discarding supernatant, cells were suspended in DMEM/F12 medium supplemented with 10% FBS and 1% antibiotics and antimycotics (Invitrogen Corp). These mammary tumor cells (~1.0 × 10^6 ^cells/plate) were seeded in tissue culture dishes and kept in a 37°C humidified atmosphere containing 95% air and 5% CO_2_. The media was changed twice a week to maintain cells in culture. Each line was passaged approximately 20 times before stability was assumed.

### Soft agar cloning assays

Soft agar cloning was performed as described previously [35] with some modification. The bottom agar was prepared with a mixture of 1.6 ml of 1 × DMEM/F12 (complete medium), 3.2 ml of 2 × DMEM/F12 (complete medium), and 3.2 ml of 1.25% Noble agar (Sigma Co, St Louis, MO, USA) and maintained at 42°C. From this, 2 ml was pipetted into each well of six-well cell culture plates and agar was allowed harden in the hood. For each well, top agar was a mixture of 0.2 ml of 1 × DMEM/F12, 0.4 ml of 2 × DMEM/F12, and 0.4 ml of 0.95% Noble agar. Five thousand cells (in 80 μl complete medium) were added into the top agar mixture. After vortexing gently, the cell containing top agar was added in a drop-wise fashion onto the bottom agar containing six-well plates (in triplicate per cell line). After resting for 10 min in the hood, the six-well plates were cultured in a 37°C incubator for 3 weeks. Colony counts were obtained under an inverted microscope, from three wells per cell line counting all colonies >50 μM in diameter.

### Doubling time in culture

Measurement of cell growth rate in culture was determined using sulforhodamine B (SRB; Sigma Co) assays as previously described [35]. Two thousand cells were seeded into each well of a six-well plate with complete medium. Cells were fixed with 50% trichloroacetic acid (TCA) at 24 h intervals for 3 days. TCA-fixed cells were then stained with 0.4% SRB for 30 min followed by three washes. Protein-bound dye was dissolved in 10 mM Tris base. Plates were read at 565 nM using a micro-plate reader. Cell-doubling time was calculated based on proliferation curves that resulted from changes in SRB absorbance over time. Data represent the means of at least three independent experiments.

### Cell proliferation assay

A CellTiter96™ AQ non-radioactive cell proliferation kit (Promega Corp, Madison, WI, USA) was used to determine the responsiveness of cells to various growth factors. Cells were plated onto 96-well plates, 5,000 cells/well for each cell line. Twenty-four hours later, the culture media were replaced by 0.5% FBS in DMEM/F12 fresh medium or the same medium containing 25 ng/ml HRG (R&D Systems, Inc, Minneapolis, MN, USA), 10 ng/ml EGF (Sigma Chemical Co), or 40 ng/ml IGF-1 (R&D Systems, Inc) for another 72 h incubation with 5% CO_2 _at 37°C. After reading at 490 nM with the micro-plate reader, the percentages of viable cells were determined by reduction of MTS (3-(4,5-dimethylthiazol-2-yl)-5-(3-carboxymethoxyphenyl)-2-(4-sulfophenyl)-2H-tetrazolium; inner salt) relative to controls. Data reflect the means of at least three independent experiments.

### RT-PCR and DNA sequencing analysis

RT-PCR analyses were performed as previously described [37]. The primers specific for rat *neu *were synthesized according to the literature [38]. Forward primer AB2913, 5'-CGG AAC CCA CAT CAG GCC-3' and reverse primer AB1310, 5'-TTT CCT GCA GCA GCC TAC GC-3' amplify the region corresponding to nucleotides 1492 to 2117 of rat *neu *cDNA [38]. The PCR products purified from agarose gel using QIAquick Gel Extraction Kit (Qiagen, Inc, San Valencia, CA, USA) were submitted to the core facility at the Oklahoma Medical Research Foundation for direct sequencing.

### Immunohistochemistry

Immunohistochemical staining of mammary tumor tissues was performed as previously described [39]. Briefly, after deparaffinization and rehydration, tissue sections were steamed in a 10 mM citrate buffer, pH 6.0, for 30 min. Non-specific reactivity was blocked with 0.3% H_2_O_2 _in buffer. For erbB3 immunoassays, CAS Block (Zymed Laboratories, Inc, South San Francisco, CA, USA) and 10% normal horse serum (Vector Laboratories, Inc, Burlingame, CA, USA) were used sequentially. For phospho-Akt immunostaining, we used 1% H_2_O_2 _and 5% normal goat serum (Vector Laboratories, Inc) sequentially. Primary antibodies included an anti-erbB2 (reactive with rat c-*neu*/erbB2 rabbit polyclonal, dilution 1:1000 (Dako, Carpinteria, CA, USA) for 2 h incubation at room temperature), anti-erbB3 (cross-reacts with mouse and human, mouse monoclonal, dilution 1:50 (NeoMarkers, Inc, Fremont, CA, USA), overnight incubation at 4°C), anti-phospho-Akt (rabbit polyclonal, diluted in 5% normal goat serum 1:12.5 (Cell Signaling Technology, Beverly, MA, USA), overnight at 4°C), or anti-phospho-MAPK (E10 monoclonal antibody, diluted in 5% normal goat serum 1:25 (Cell Signaling Technology), overnight at 4°C). After multiple washes with buffer, tissue sections were sequentially incubated for 30 min at room temperature with diluted biotinylated secondary antibody (1:500; Dako) and VECTASTAIN Elite ABC reagent (Vector Laboratories, Inc) diluted in PBS. After reaction with diaminobenzidine (Dako) and counterstaining with hematoxylin, tumors were individually examined. Each slide was evaluated in its entirety for antigen expression, cell type and histopathological diagnoses.

### Immunoprecipitation and Western blot analysis

Immunoprecipitation and Western blot assays were performed as previously described [40]. Briefly, cells were lysed in NP-40 lysis buffer (50 mM Tris-HCl, pH 7.4, 150 mM NaCl, 0.5% NP-40, 50 mM NaF, 1 mM Na_3_VO_4_, 1 mM phenylmethylsulfonyl fluoride, 25 μg/ml leupeptin, 25 μg/ml aprotinin). The supernatants were cleared by centrifugation. Protein concentrations were measured using the Coomassie plus protein assay reagent (Pierce Chemical Co, Rockford, IL, USA). Total cell lysates containing 200 μg of protein were subjected to immunoprecipitation in the presence of 1 μg anti-erbB2 antibody (mouse monoclonal antibody, Ab-4; Oncogene Science Products, Cambridge, MA, USA) for 2 h at 4°C, followed by incubation with immobilized protein A-agarose (Roche Diagnostics Corp, Indianapolis, IN, USA) at 4°C overnight with rotation. For Western blot analyses, the immunoprecipitates or equal amounts of crude extracts were boiled in Laemmli SDS-sample buffer, resolved by SDS-polyacrylamide gel electrophoresis, transferred to nitrocellulose (Bio-Rad Laboratories, Hercules, CA, USA), and probed with different primary antibodies. After the blots were incubated for another 1 h at room temperature with horseradish peroxidase-labeled secondary antibody (goat anti-rabbit IgG or goat anti-mouse IgG; Perkin Elmer, Boston, MA, USA), the signals were detected using the Enhanced Chemiluminance assay (Amersham Life Science Inc., Arlington Heights, IL, USA) according to the manufacturer's instructions.

## Results

### Co-expression of erbB2 and erbB3 protein in tumor-derived cell lines and tumors

**Figure 1:**
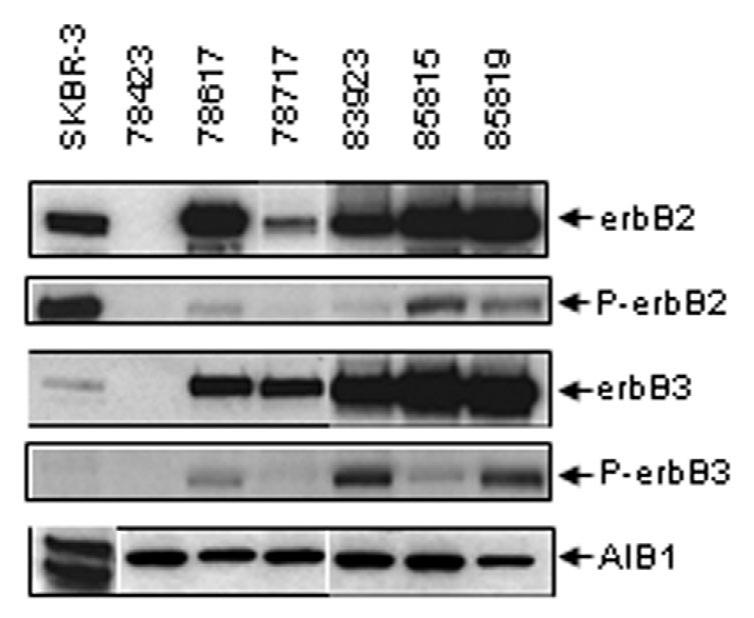
Protein expression of erbB2, P-erbB2, erbB3, P-erbB3 and AIB1 in breast tumor cell lines. The cell lysates from human breast cancer cell line SKBR-3 and the six novel, mammary tumor-derived cell lines were prepared as described in Materials and methods. 50 μg total cell lysates were used for Western blot analyses with antibodies directed against erbB2 (c-*neu *Ab-3, Oncogene Research Products), P-erbB2 (clone PN2A, NeoMarkers, Inc), erbB3 (Ab-7, NeoMarkers, Inc), P-erbB3 (clone 21D3, Cell Signaling Technology) and AIB1 (clone 34, BD Biosciences Pharmingen, San Diego, CA, USA).

Western blot analyses were used to determine erbB2 and erbB3 protein expression in tumor-derived cell lines (and the control SKBR-3 human breast cancer cell line). The majority of tumor-derived cell lines expressed moderate to high levels of both erbB3 and erbB2 (Fig. 1). In general, lines with the highest erbB2 expression showed the highest levels of erbB3 protein. Tyrosine phosphorylation (activation) of these receptors was examined by Western blots using antibodies specific for phophorylated erbB2 (P-erbB2) or phosphorylated erbB3 (P-erbB3). Tumor lines with co-overexpression of both proteins showed higher P-erbB2 and P-erbB3 levels (Fig. 1). The intensity of P-erbB2 and P-erbB3 signals did not necessarily correlate with their corresponding protein levels. The expression of either receptor protein was undetectable in only one of our novel, derived tumor cell lines (78423). AIB-1 (also called SRC3, RAC3, ACTR and p/CIP), a co-activator of estrogen receptor commonly amplified in breast cancer cells [41], was used as a loading control. Expression of AIB-1 further established the origin of these cells as mammary-derived.

To confirm the transformed characteristics of these lines, soft agar cloning assays (which quantitate anchorage-independent cloning capability) were used. All six tumor-derived cell lines formed colonies in soft agar. Colony formation was variable when comparing one cell line with another (range 17–180, Table 1). There was no correlation between the ability of a cell line to form anchorage-independent clones and the expression levels of erbB2 or erbB3.

**Figure 2:**
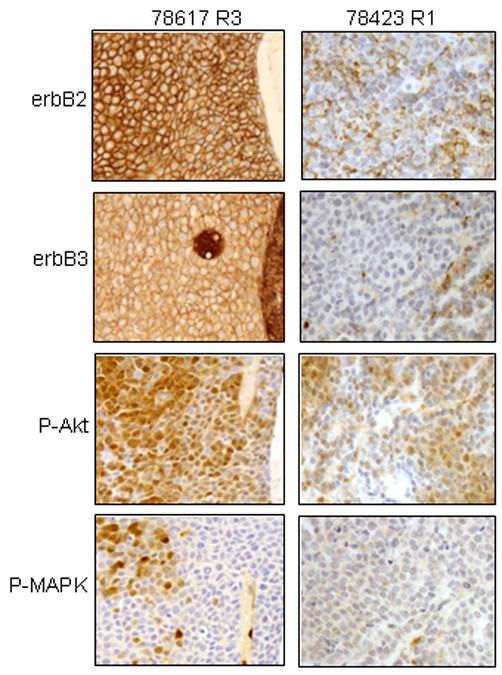
Immunohistochemical staining for erbB2, erbB3, phospho-Akt (P-Akt) and phospho-MAPK (P-MAPK) in mammary tumor tissues. Representative photomicrographs were taken from the similar area of 78617 R3 and 78423 R1 mammary tumor sections (40X).

Immunohistochemical methods were used to visualize RTK expression and downstream signaling (protein activation) by tumors *in situ*. Tumors showed strong and typically diffuse co-expression of both erbB2 and erbB3. The only exception to this was the mammary tumor 78423 R1, the progenitor of the cell line that did not co-express erbB2 and erbB3 discussed above. We also studied RTK signaling activation *in situ*, using phosphospecific antibodies. Phosphorylated-Akt (P-Akt) showed cytoplasmic and membranous staining, which was less diffuse than the erbB-2 expression. Phosphorylated-MAPK (P-MAPK) was the most selectively expressed, typically expressed by clustered or isolated tumor cells as shown in Fig. 2 (left panel) with tumor 78617 R3. The majority of tumor cells from 78423 R1 were erbB3 negative, although some cells showed weak erbB2 protein expression. In this later tumor, P-Akt staining was weak with clustered or isolated tumor cells and no staining for P-MAPK was observed (Fig. 2, right panel). The histological, cytological and biological features of these tumors have been reported elsewhere [36,42]. As a control, we also studied cytokeratin expression and all tumors were positive. This confirmed the epithelial nature of these tumors (data not shown).

### Sequencing analyses of the transgene *neu *in established mammary tumor-derived cell lines

**Figure 3:**
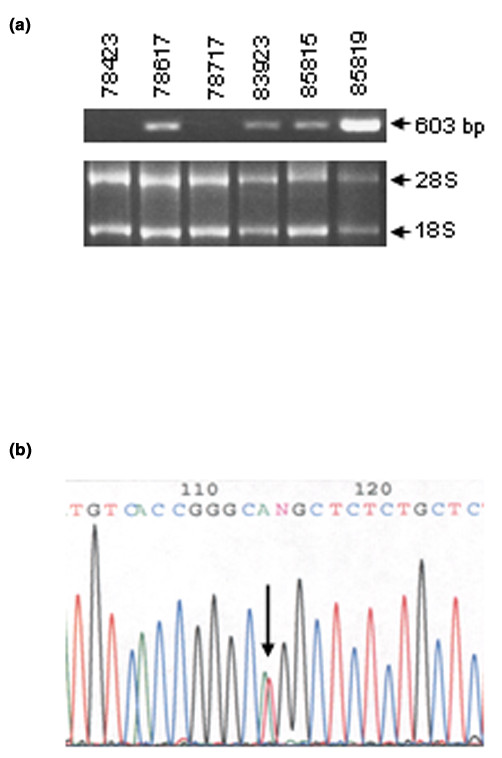
RT-PCR and sequencing analyses of partial extracellular domain of rat *neu *in the tumor-derived cell lines. **(a) **RT-PCR analyses of rat *neu*. Total RNA isolated from the indicated tumor cells by the TRIZOL reagent was analyzed with electrophoresis using a 1% agarose gel containing ethidium bromide and visualized under UV light (bottom). First-strand cDNA was synthesized using a kit from Roche Diagnostics Corp. The partial extracellular domain of rat *neu *was amplified with specific primers. The PCR products were separated on a 1.2% agarose gel containing ethidium bromide and visualized under UV light (top). **(b) **Partial sequencing of the PCR product from 83923 cells with reverse primer AB1310. The mixture of wt nucleotide T (red) and mutant nucleotide A (green) is indicated by an arrow.

As alluded to above, in-frame deletions of 7–12 amino acids have been reported in the extracellular region of the transgene, proximal to the transmembrane domain [38]. To study the mutational status of tumor-derived lines, we performed RT-PCR amplification of exactly the same region followed by direct sequencing analysis. The PCR primers used were specific for rat *neu *and were designed to amplify the 603 bp extracellular region [38]. Of six tumor-derived cell lines used in this manuscript and therefore studied for mutation, only four showed PCR gene amplification (Fig. 3a). Of these, the strongest PCR signal was seen in 85819 cells. These data are consistent with our Western blot results that showed overexpression of the rat *neu*/erbB2 in only the four PCR-positive lines (Fig. 1). Direct sequencing of the PCR products revealed no deletion mutations in the amplified product. Sequencing showed three of the four were wt rat *neu *cDNA sequence. Sequencing data from the 83923 cells indicated a mixture of two kinds of *neu *cDNA. Using a reverse primer, we verified that both wt and point mutation *neu *transcripts co-existed in 83923 cells (Fig. 3b). This suggests biclonal populations or a heterozygous mutation. Further studies and sub-cloning are in process.

### Mammary tumor cell response to growth factors corresponds with erbB receptor data

**Figure 4:**
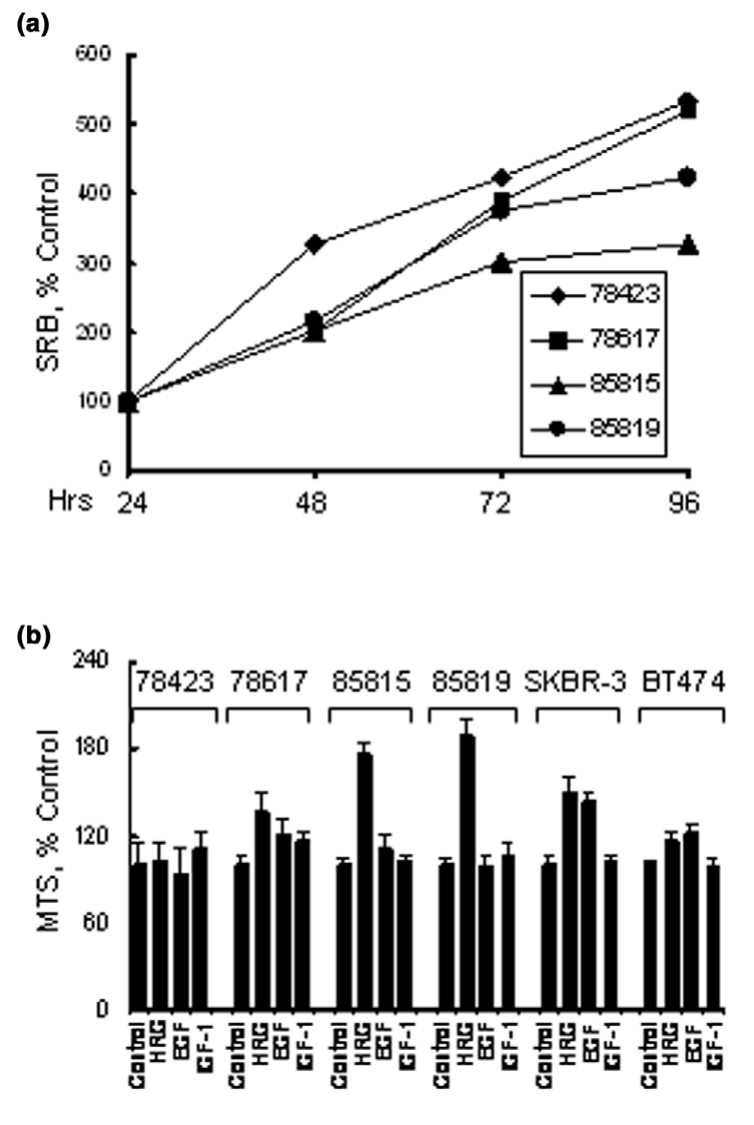
Proliferation of the tumor-derived cell lines and their responsiveness to growth factors. **(a) **The tumor-derived cell lines 78423, 78617, 85815 and 85819 were subjected to SRB assays for measurement of cell growth rate as described in Materials and methods. The means of at least three independent experiments were plotted. SD for each point was less than 10%. **(b) **The indicated breast cancer cells (5 × 10^3^) in 0.1 ml culture media were plated onto 96-well plates. After 24 h incubation, cells were grown in either 0.1 ml fresh medium with 0.5% FBS as control, or 0.1 ml same medium containing either 25 ng/ml HRG or 10 ng/ml EGF, and 40 ng/ml IGF-1. Cells were incubated at 37°C with 5% CO_2 _for another 72 h, and the percentages of surviving cells from each group relative to controls, defined as 100% survival, were determined by reduction of MTS. Data reflect the means of at least three independent experiments.

To study the functionality and interactions of the erbB receptors, 78423 and other three representative mouse mammary tumor-derived lines with the highest expression of wt erbB2 and co-expression of erbB3 were chosen for further study. Baseline proliferation was determined using monolayer culture conditions and the SRB assay (Fig. 4a). Some variability in the basal doubling time was observed between these cell lines. The mouse mammary tumor cell lines 78423, 78617, 85815 and 85819 showed population doubling times of 15.15 ± 1.10, 16.25 ± 1.40, 30.85 ± 2.31 and 20.35 ± 1.89 h, respectively. Using an MTS assay, we then tested the response of these lines to EGF, HRG and insulin-like growth factor (IGF)-1 (Fig. 4b). HRG strongly stimulated the proliferation of three of the four mouse mammary tumor cell lines (78617, 85815, 85819) with overexpression of both erbB2 and erbB3. Proliferation was not induced by EGF or IGF-1, which bind to EGFR and IGF-1 receptor, respectively. HRG also promoted the growth of SKBR-3 and BT-474 human breast cancer cells (controls). These data strongly support a functional interaction between the wt-rat *neu*/ErbB2 and endogenous mouse erbB3.

### HRG activation of PI-3K/Akt and MAPK kinase (MEK)/MAPK signaling promotes mammary tumor cell growth

**Figure 5:**
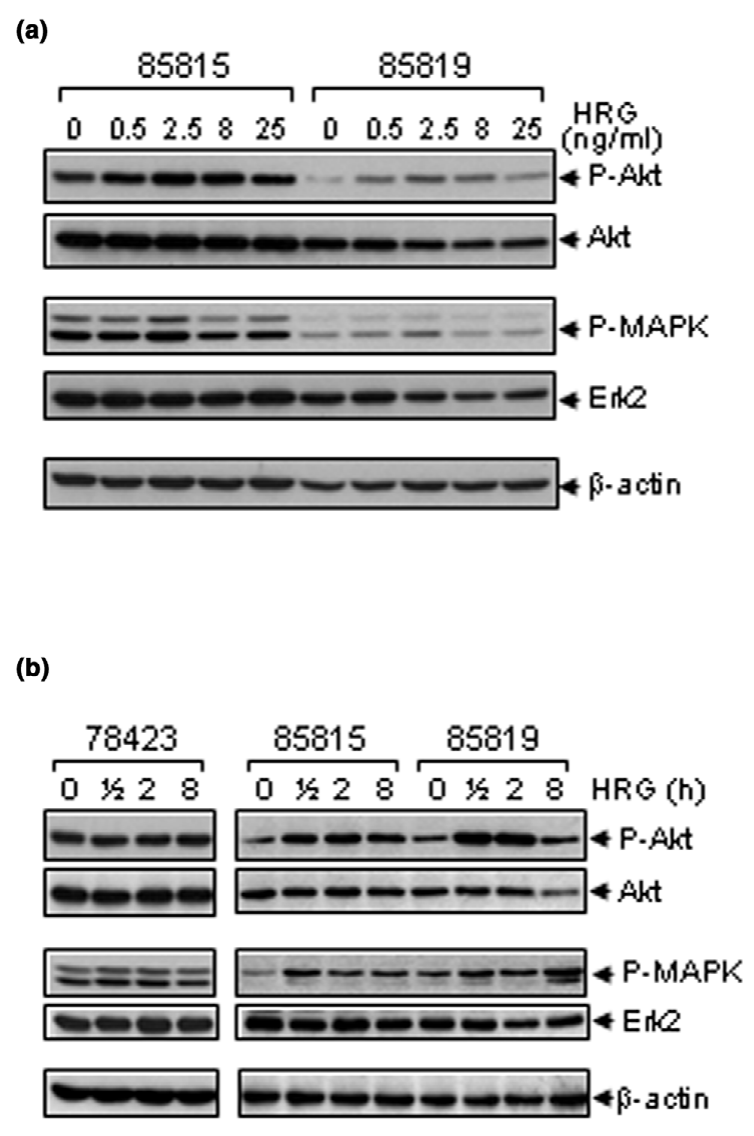
Effects of HRG on the phosphorylation of Akt and MAPK in tumor-derived cells. **(a) **85815 and 85819 cells were cultured overnight in medium containing 0.5% FBS before being exposed to HRG at the indicated concentrations for 2 h. Cells were harvested and 50 μg total cell lysates were subjected to Western blot analysis for total Akt, phosphorylated Akt, total ERK2 (polyclonal antibody C-14; Santa Cruz Biotechnology, Inc, Santa Cruz, CA, USA), and phosphorylated MAPK (E10 mAb; Cell Signaling Technology) expression. β-actin was used as loading control. **(b) **78423, 85815 and 85819 cells were cultured overnight in medium containing 0.5% FBS before being exposed to 2.5 ng/ml HRG for the indicated time intervals. At each time point, cells were harvested and 50 μg total cell lysates were subjected to Western blot analysis for total Akt, phosphorylated Akt, total ERK2, and phosphorylated MAPK expression. β-actin was used as loading control.

It is well documented that the MEK/MAPK and PI-3K/Akt pathways are the two major signal transduction pathways downstream of the erbB receptors [11,17-19]. To determine which signaling pathways were activated in the mouse-derived mammary tumor cells exposed to HRG, we performed Western blots to detect P-MAPK or P-Akt. With 2 h of HRG treatment, both P-Akt and P-MAPK increased in the 85815 and 85819 mouse mammary tumor cell lines (Fig. 5a). This study included a series of HRG concentrations, and stimulation was maximal at a concentration of 2.5 ng/ml. Next, we performed a time-course analysis to further verify these results. HRG stimulated both Akt and MAPK in 85815 and 85819 cells, whereas it had no effect on Akt or MAPK activation in the 78423 cells (Fig. 5b). These data were consistent with the results of minimal stimulation by HRG in this cell line (Fig. 4b). In aggregate, these data suggest that HRG induces activation of both MEK/MAPK and PI-3K/Akt signaling transduction pathways in mammary tumor cells with elevated expression levels of both the transgene rat c-*neu/ErbB2 *and the endogenous mouse *ErbB3 *gene. This activation was both dose- and time-dependent.

To study cross-species functional interactions between the rat c-*neu/Erb*B2 transgene and mouse *ErbB3*, we evaluated tumor and tissue expression *in vivo*, ligand-associated interactions, and signaling *in vitro*. Immunohistochemical studies showed cytoplasmic P-Akt and P-MAPK expression in tumor cells with erbB2 and erbB3 co-expression, predominantly a perivascular distribution. In rare tumors without erbB2 and erbB3 expression (e.g. 78423 R1), the perivascular distribution was not identified and only rare cells showed immunoreactivity. This evidence of perivascular pathway activation suggests that ligand-associated signaling via erbB3 may be involved. Ligand-associated signaling probably provides enhanced growth or pro-tumorigenic signaling, in addition to ligand-independent, transgene activation. Our data, and those from others showing frequent erbB3 upregulation in transgenic mice bearing activated *neu/ErbB2*, suggest that the concomitant upregulation of erbB3 and ligand-associated signaling may be an important additional factor in both wt and activated *neu/ErbB2*-associated mammary tumor development. To further define the role of HRG (ligand)-associated signaling, we utilized derived cell lines and specific inhibitors *in vitro*. The PI-3K inhibitor LY294002 was significantly more potent than the MEK inhibitor PD98059 in blocking the stimulatory effects of HRG (Fig. 7). Hence, while the MEK/MAPK and PI-3K/Akt signaling cascades both contribute HRG induced proliferation, the PI-3K/Akt pathway appears to provide the dominant response.

### Physical interaction between wt-rat-c-*neu*/ErbB2 and endogenous mouse erbB3

The erbB2/erbB3 complex is believed to be the most biologically active erbB heterodimer [43,44], with potent activation of the downstream signaling cascade [13,14]. Since both erbB2 and erbB3 were highly expressed by our mammary tumor cell lines and HRG-promoted tumor cell proliferation, we sought physical evidence that the wt-rat-*neu*/ErbB2 could form a complex with the endogenous mouse erbB3. Immunoprecipitation of erbB2, followed by Western blot analysis for erbB2 and erbB3 (Fig. 8a) showed a low level of complex formation between these receptors in untreated cell lines. HRG treatment significantly increased the physical interaction between the rat transgene and mouse erbB3 in two out of three cell lines. The antibody we used for immunoprecipitation (c-*neu *Ab-4) appeared to be wt-rat-*neu/*ErbB2-specific, because human erbB2 was not immunoprecipitated from SKBR-3 cell lysates (Fig. 8a, upper panel), although it was expressed by SKBR-3 cells (Fig. 8b, Western blot analysis with c-*neu *Ab-3). HRG treatment (for 2 h) did not increase the total protein levels of erbB2 or erbB3 as compared with untreated cell lines (Fig. 8b).

## Discussion

We have shown that transgenic mice bearing the wt-rat c-*neu *gene, under control of the MMTV promoter, develop mammary tumors that overexpress the rat c-*neu *transgene [45,46] and the endogenous mouse erbB3 protein, in the vast majority of cases. We have shown a functional interaction between these two important RTK receptors and a role for ligand-induced signaling *in vitro *and *in vivo*. While others have reported that transgenic mice bearing activated forms of rat c-*neu*/*erb*B2 have co-expression of erbB2 and endogenous erbB3 in mammary tumors [32], direct physical and functional interactions between these two species receptors have not previously been reported.

Deletion mutants of the *neu *oncogene have been reported in two out of three of the mammary tumors derived from this wt-rat c-*neu *transgenic model [38]. We did not find the same mutation rate or type in selected tumor-derived cell lines. However, we have identified a potential point mutation in 83923 cells (Fig. 3). This missense mutation is located inside the same extracellular region of *neu *where the deletion mutations have been reported. This particular mutation changes the amino acid 654 serine (codon AGC) into cysteine (codon TGC). It is different from the active *neu *mutation G664V reported in the transmembrane domain [47]. The biological significance of the newly discovered S654C mutant *neu *is not yet known.

Using ligand stimulation with or without specific inhibitors, we have studied RTK-induced signaling in response to HRG and have shown activation of both PI-3K/Akt and the MEK/MAPK signal transduction pathways. A greater role for PI-3K/Akt signaling was suggested in response to HRG treatment (Fig. 7). PI-3K/Akt signaling is known to be regulated by erbB2-mediated tyrosine kinase activity. This pathway plays a crucial role in cell proliferation and survival [18] and has been associated with the pathogenesis of human breast cancers. PI-3K/Akt activation has also been cited as a key pathway that influences chemo-resistance patterns [48,49]. Akt is frequently upregulated in *ErbB2 *amplified or overexpressing human breast cancer cells. These similarities between our transgenic model and human breast carcinogenesis suggest that the model and derived tumor cell lines may be a useful resource to study ligand dependent and independent RTK signaling *in vivo *and *in vitro*.

**Figure 6:**
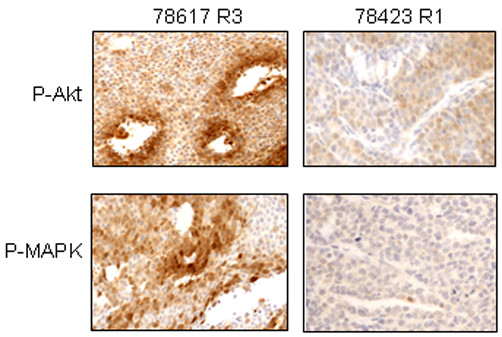
Immunohistochemical staining for phospho-Akt (P-Akt) and phospho-MAPK (P-MAPK) in 78617 R3 and 78423 R1 mammary tumor tissues. Procedure used was similar to Fig. 2. Representative photomicrographs were taken from perivascular areas of the tumor sections (20X).

**Figure 7:**
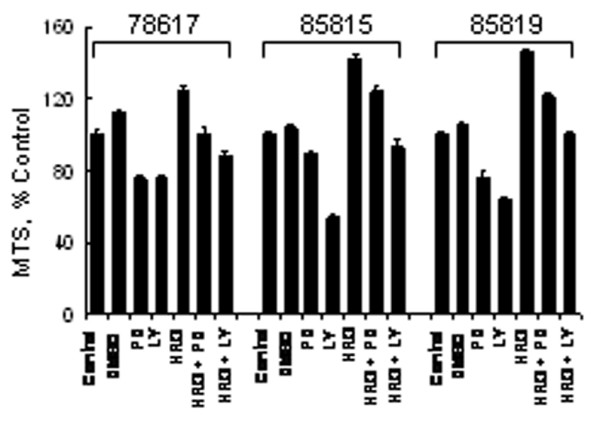
Inhibitory effects of PD98059 and LY294002 blocking HRG-mediated tumor cell proliferation. The indicated breast cancer cells (5 × 10^3^) in 0.1 ml culture media were plated onto 96-well plates. After 24 h incubation, cells were grown in either 0.1 ml fresh medium with 0.5% FBS as control, or 0.1 ml same medium containing PD98059 (6.7 μM for 78617 and 85815, 5 μM for 85819), or LY294002 (3.3 μM for 78617 and 85815, 2.5 μM for 85819), or 25 ng/ml HRG alone or in combination of the same concentrations of HRG and PD98059, or HRG and LY294002. Cells were incubated at 37°C with 5% CO_2 _for another 72 h, and the percentages of surviving cells from each group relative to controls, defined as 100% survival, were determined by reduction of MTS. Data reflect the means of at least three independent experiments.

**Figure 8:**
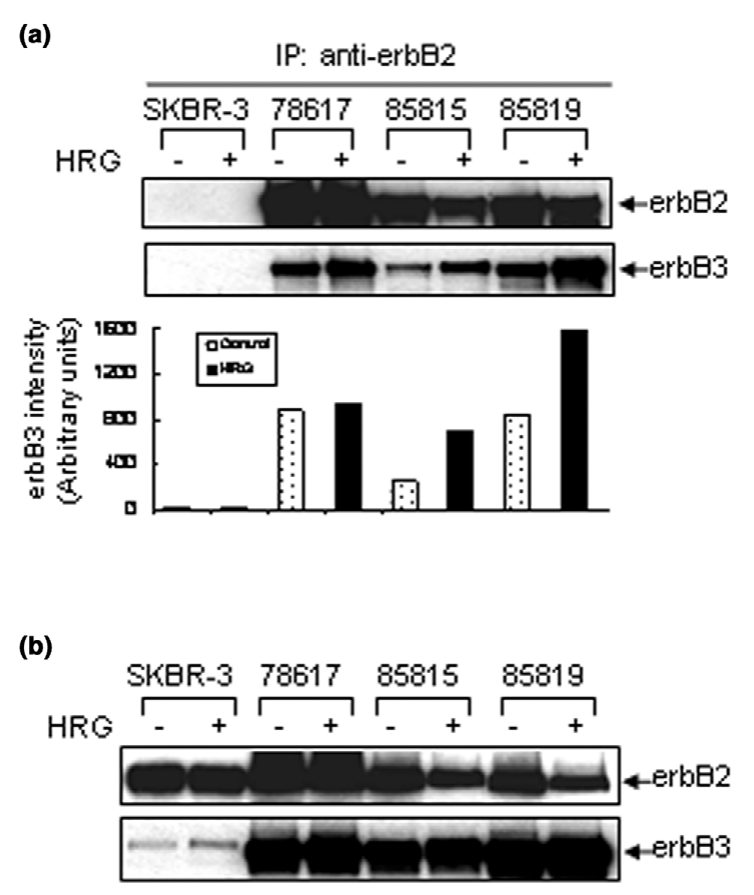
Increased physical associations between wt-rat c-*neu *protein and mouse erbB3 by HRG treatment. **(a) **The indicated breast cancer cells were cultured overnight in medium containing 0.5% FBS, and then incubated with or without 2.5 ng/ml HRG for another 2 h. Cell lysates were prepared and 200 μg of total protein was subjected to immunoprecipitation with anti-erbB2 specific antibody (c-*neu *Ab-4), followed by Western blot analysis for erbB2 (c-*neu *Ab-3) and erbB3. The bar graph underneath was obtained by densitometry analysis. The relative signal intensities of erbB3 were measured by EAGLE EYE™ II (Stratagene, La Jolla, CA, USA). **(b) **The same bath of 50 μg total cell lysates was subjected to Western blot analysis with antibodies directed against erbB2 and erbB3.

As a major ligand for erbB3, HRG is known to bind to erbB3, foster heterodimer complex formation and promote potent downstream signaling [12]. HRG can thus promote mammary tumorigenesis, cell growth, differentiation and phenotypic aggression [50]. Our immunohistochemical studies of tumors for phosphorylated proteins facilitated studies of the cellular location and architectural context of signaling. We noted enhanced phosphorylated Akt and MAPK in a perivascular distribution in mammary tumors, with overexpression of both erbB2 and erbB3 (Fig. 6), suggesting that circulating HRG may enhance the physical and functional erbB2/erbB3 interactions *in vivo*, similar to what we observe *in vitro*. This study has focused primarily on erbB3, whereas others have demonstrated upregulation of EGFR in tumors (by immunohistochemistry and Western blot) in the same model system [33]. Low and variable expression of EGFR has also been found in mammary tumors that develop in transgenic mice bearing activated forms of rat c-*neu*/ErbB2 [32]. Using *in vitro *analyses of the tumor-derived cell lines, we have found no significant physical or functional interaction between EGFR (erbB1) and erbB2 in the presence of EGF (data not shown). However, by immunohistochemical study, we also detected erbB1 expression at the tumor periphery as reported by DiGiovanna [33]. These data suggest to us that erbB3 plays a more significant role in tumorigenesis than erbB1 in this model system.

These data and this model probably have relevance to human breast cancer biology and treatment strategies. We have reported that only a minority of erbB2-altered invasive human breast cancers have overexpression of erbB1 (EGFR) and activation of erbB2 [51]. Given the complexity of the RTK receptors, various ligands and downstream signaling, it is likely that combinations of these factors including erbB3 contribute to cell signaling, biological behavior and treatment response [52,53]. To date, the role of erbB3 in human breast carcinogenesis is not well defined, although many investigators have suggested that HRG-associated signaling may be important. In view of these complexities, it is not surprising that erbB2 aberrant breast cancers have shown variable responses to anti-erbB2 therapeutics [52,53]. It is widely believed that co-expression of other erbB RTK family members may be one mechanism of Herceptin resistance [54]. Ligand-induced heterodimerization between erbB3 and erbB2, the most potent signaling complex amongst the various heterodimers, is one likely mechanism of Herceptin resistance [55]. More detailed investigations using banks of human tumors and clinical trial-associated specimens, to define the incidence of erbB3 abnormalities, functional complex formation and downstream signaling, may provide important new clues regarding these interactions and their role in breast carcinogenesis.

## Conclusion

Our results indicate that over-expression of endogenous mouse erbB3 plays an important role in the development and progression of mammary tumors that arise in mice bearing the wt-rat c-*neu *transgene. The functional and physical interactions between these important cross-species erbB receptors result in activation of both PI-3K/Akt and MEK/MAPK signaling. These data support the concept that ligand-dependent and -independent signaling through erbB2 may promote mammary tumorigenesis in these transgenic mice, similar to what is observed in human breast cancers.

## Abbreviations

DMEM = Dulbecco's modified Eagle's medium; EGF = epidermal growth factor; EGFR = EGF receptor; ER = estrogen receptor; FBS = fetal bovine serum; HRG = heregulin; IGF-1 = insulin-like growth factor-1; mAb = monoclonal antibody; MAPK = mitogen-activated protein kinase; MEK = MAPK kinase; MMTV = mouse mammary tumor virus; PBS = phosphate-buffered saline; RTK = receptor tyrosine kinase; PI-3K = phosphoinositide 3-kinase; RT-PCR = reverse transcription-polymerase chain reaction; SRB = sulforhodamine B; TCA = trichloroacetic acid; TGF-α = transforming growth factor-α.

## Competing interests

The author(s) declare that they have no competing interests.

## Authors' contributions

The authors' contributions to this research work are reflected in the order shown, with the exception of ADT who supervised the research and finalized the report. AK, BL and DOE carried out most of the experiments. AK and BL drafted the manuscript. KMA collected mammary tumors from the transgenic mice. LDJ performed immunohistochemistry analysis. CM maintained tumor cell culture. SME, XY and ADT conceived the study and participated in its design and coordination. All authors read and approved the final manuscript.

## Figures and Tables

**Table 1:** Anchorage-independent cloning of mammary tumor-derived cell lines

Cell Lines	SKBR-3^a^	78423	78617	78717	83923	85815	85819
Colonies in soft agar^b^	422	87	49	24	41	180	17

^a^Human breast cancer cell line SKBR-3 was used as positive control.

^b^Colony numbers represent average of triplicates for each cell line.
